# A novel prognostic immunoscore based on The Cancer Genome Atlas to predict overall survival in colorectal cancer patients

**DOI:** 10.1042/BSR20210039

**Published:** 2021-10-19

**Authors:** Zuxiong Tang, Yufan Wu, Ding Sun, Xiaofeng Xue, Lei Qin

**Affiliations:** 1 Department of General Surgery, The First Affiliated Hospital of Soochow University, Suzhou 215000, JiangSu Province, China; 2 Department of Surgery, Kunshan Hospital of Traditional Chinese Medicine, Kunshan 215300, JiangSu Province, China

**Keywords:** colorectal cancer, Overall survival, Prognostic immunoscore, The Cancer Genome Atlas

## Abstract

Colorectal cancer (CRC) is highly prevalent worldwide. The relationship between the infiltration of immunocytes in CRC and clinical outcome has been investigated in recent years. The present study aims to construct a new prognostic signature using an immunocyte panel. Our novel prognostic immunoscore included 13 types of immunocytes, which were identified by least absolute shrinkage and selection operator (LASSO)-Cox regression. The time-dependent receiver operating characteristic (ROC) curve and Kaplan–Meier survival estimates were applied to evaluate the prognostic ability. Compared with the signature based on a single immune marker (i.e., *CD8* mRNA expression and CD8^+^ expressing T cells), the novel prognostic immunoscore possessed better specificity and sensitivity of prognosis (area under the curves (AUCs) are 0.852, 0.856, and 0.774 for 1-, 2-, and 3-year survival times, respectively). Significant differences were identified between the high and low immunoscore groups in overall survival and disease-free survival in training and validation cohorts. Combining the immunoscore with clinical information may provide a more accurate prognosis for CRC. The immunoscore can identify patients with poor outcomes in the high Tumor Mutational Burden (TMB) group, who may benefit the most from immunotherapy. The immunoscore was also closely related to two immune checkpoints (i.e., PD-L1 and PD-1, r = 0.3087 and r = 0.3341, respectively). Collectively, our study demonstrates that the novel prognostic immunoscore reported here may be useful in distinguishing different prognoses and may improve the clinical management of patients with CRC.

## Introduction

Colorectal cancer (CRC) is highly prevalent worldwide, and is the third most common type of cancer in the world, after lung and breast cancer [1]. A growing body of evidence has shown that a relationship exists between a CRC patient’s immune microenvironment and his/her prognosis [2].

In recent years, immune profiling studies have reached the forefront of cancer research [3]. Various immunocyte subtypes constitute the complex immune response to CRC [4]. The different stages and pathological types of cancer associating with the prognosis of CRC tend to activate different immunocytes’ subtypes [5]. Therefore, they may be significant for clinical research [6,7]. However, there are few studies investigating the prognostic ability of the immunocyte fraction [8].

The Cancer Genome Atlas (TCGA) [8,9] is an enormous cancer genomics project that characterizes over 20000 primary cancer tissues and molecularly matches normal tissues spanning 33 cancer categories. The creation of TCGA and the ubiquity of high-throughput sequencing data have meant that the sharing of genomic datasets related to cancer research has become more common.

CIBERSORT [10] is a new method that can estimate the compositions of 22 immunocyte categories based on the TCGA gene expression data. In our study, we paid close attention to the immunocyte fraction and the novel prognostic immunoscore based on the immunocyte subtypes for survival analysis and further research [11].

## Materials and methods

### Sources of expression profiles and clinical data

We obtained a dataset containing mRNA expression profiles from paired CRC tissues and adjacent non-tumorous tissues. As of 1 January 2018, this dataset, which was acquired from TCGA (https://portal.gdc.cancer.gov/), contained 441 CRC tissue samples and 44 adjacent non-tumorous tissue samples. All sequencing procedures were performed on the Illumina HiSeq platform. Accompanying clinical data (‘id’, ‘survival time’, ‘survival state’, ‘stage’, ‘lymph node involvement’, ‘sex’, ‘age’, and ‘tumor site’) were obtained from the University of California at Santa Cruz Xena browser (UCSC, https://xenabrowser.net/datapages/?dataset). All data were obtained from TCGA or the UCSC browser. Because the initial deposition of the data abided by the ethical guidelines of TCGA platform, there was no need to obtain additional ethical clearance from our local research ethics committee.

### Estimation of immunocyte subtypes

The CIBERSORT method and the Leukocyte signature matrix 22 (LM22), which contains 547 genes and allows highly sensitive and specific discrimination of 22 human immunocyte subtypes, were used to quantify the proportions of immunocytes, including B cells, T cells, natural killer cells, macrophages, dendritic cells, and myeloid subgroups in the CRC samples. The reliability of the CIBERSORT method was verified by microarray gene data. This method used Monte Carlo sampling to derive a *P*-value for the deconvolution of each sample, which was well designed to provide a reliable evaluation of the results. The proportions of immunocyte subtypes were believed to be accurate by the CIBERSORT method at a threshold of *P*<0.050. Patients meeting the threshold of *P*<0.050 were selected for further research. The sum of all estimated proportions of immunocyte subtypes was set at 1 for each sample.

### Establishment of a prognostic immunoscore

We structured the prognostic immunoscore by implementing least absolute shrinkage and selection operator (LASSO)-Cox [12] regression to analyze the dataset of immunocyte proportions. We have used the LASSO-Cox regression analysis in this previous study [13], which established a prognostic model based on an miRNA panel to better predict the survival of head and neck squamous cell carcinoma patients. We followed the methods of Xue et al., 2019 [13]. Firstly, the patients were separated into training and validation sets at a ratio of 6:4 using the stratified randomization method. Sixty percent of the patients were as`signed to the training cohort to recognize and assess biomarkers, and the remaining 40% were allotted to the validation cohort to validate the immunoscore. The optimal cut-off value for each immunocyte subtype was evaluated based on the relationship between the overall survival and cell composition in the training group by the survminer R package. Secondly, LASSO-Cox regression analysis was applied to determine the prognostic immunoscore. Finally, we evaluated the prognostic power of the immunoscore using the time-dependent receiver operating characteristic (ROC) curve [14] and Kaplan–Meier survival estimates.

### Univariate and multivariate Cox regression analyses

We performed both univariate and multivariate Cox regression analyses on the CRC samples to assess the independence of the prognosis from other clinical information (i.e., age, sex, tumor mutational burden (TMB), stage, and lymph node involvement). Hazard ratios (HRs) and 95% confidence intervals were calculated using the SPSS 19.0 software (IBM SPSS, Chicago, IL, U.S.A.). All tests performed were two-tailed.

### Pearson correlation analysis

The relationship between the immunoscore and selected immune checkpoints was determined by Pearson correlation analysis using the SPSS 19.0 software.

### Statistical analysis

Data were analyzed using SPSS 19.0 software. Statistical significance was assessd using *t* test. Probability values of less than 0.05 were considered significant. The difference of survival time between different groups was analyzed by the log-rank test.

## Results

### CRC patients’ immunocyte proportions were calculated based on data from TCGA and the CIBERSORT method

**Figure 1:**
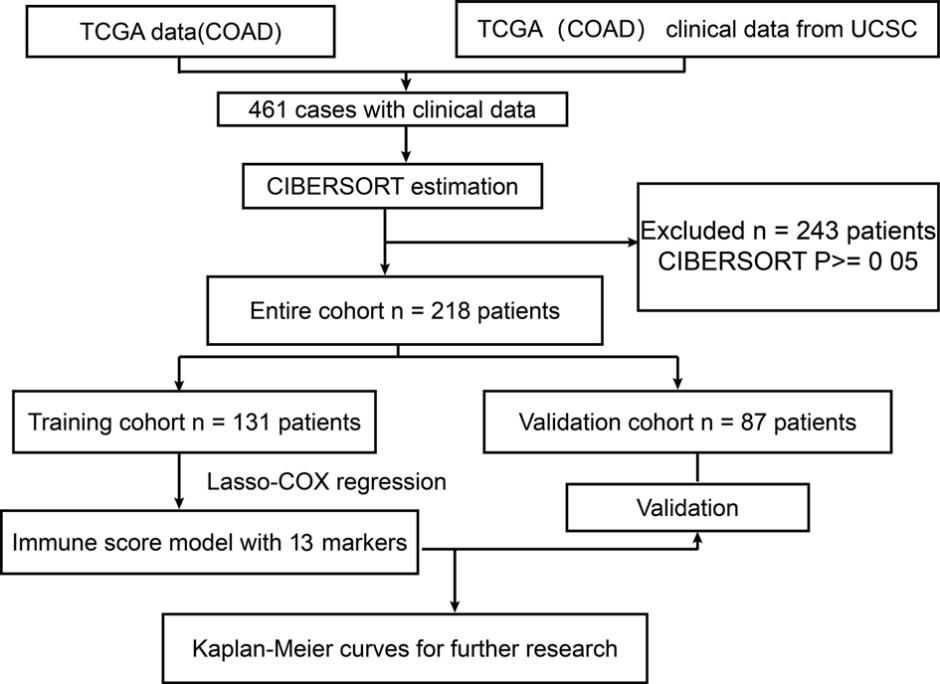
Overall workflow used to develop the immunoscore

**Figure 2:**
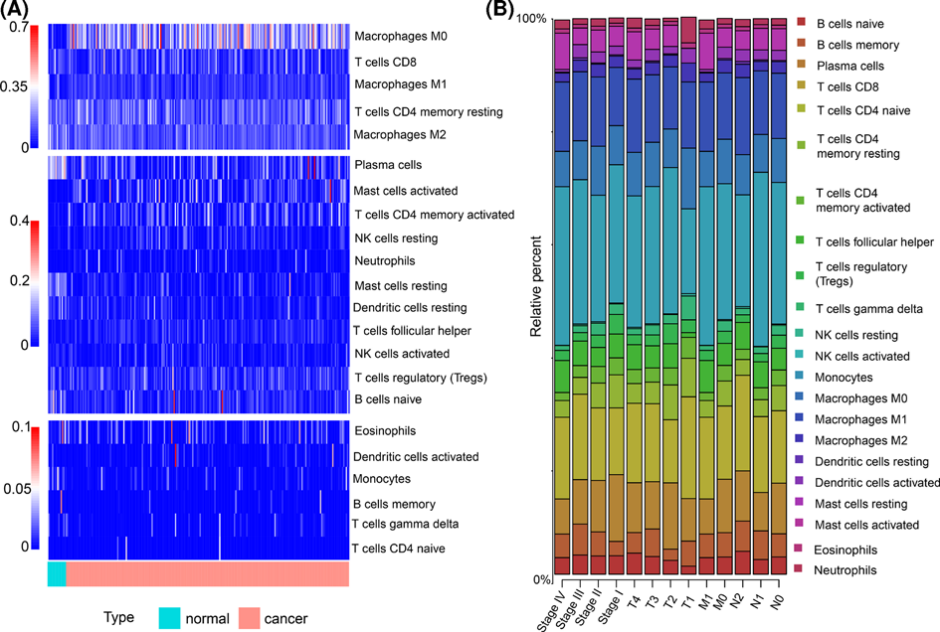
Heatmap and bar plot of 22 immunocyte subtypes in CRC patients (**A**) Heatmap of 22 immunocyte subtypes in CRC patients. Normal and CRC tissues are distinguished by blue and red labels, respectively. (**B**) Bar plot of 22 immunocyte subtypes in CRC patients. The sum of the immunocyte proportions was set at 1 for each case.

A flow chart of the analysis process is depicted in Figure 1. Two hundred and eighteen CRC patients with overall survival information were selected for further analyses after the CIBERSORT method. All patients’ clinical features are listed in Table 1. To avoid bias, we excluded cases with unknown variables from subsequent analyses. A summary of the immunocyte compositions is provided in Figure 2. The different immunocyte proportions between CRC and adjacent normal tissues are depicted in Figure 2A. The immunocyte fractions in the different subgroups of CRC patients are provided in Figure 2B. Generally speaking, the five most common immunocyte subtypes in CRC patients were M0 macrophages, CD8^+^ T cells, M1 macrophages, resting memory CD4^+^ T cells, and M2 macrophages. We observed these cell fraction patterns in all clinical subgroups.

**Table 1:** Baseline patient characteristics

Item	Number	Percent (%)
**Age**		
<65	82	37.6%
≥65	136	62.4%
**Sex**		
Male	113	51.8%
Female	105	48.2%
**Stage**		
I	34	15.6%
II	93	42.7%
III	61	28.0%
IV	22	10.1%
Unknown	8	3.7%
**Tumor site**		
Ascending colon	43	19.7%
Cecum	60	27.5%
Descending colon	6	2.8%
Transverse colon	37	17.0%
Sigmoid colon	58	26.6%
Unknown	14	6.4%
**Chemotherapy**		
Performed	69	31.7%
Unperformed	149	68.3%
** Lymphatic invasion**		
Positive	84	38.5%
Negative	117	53.7%
Unknown	17	7.8%
**Total**	218	100.0%

### The poor prognostic ability of a single immune marker (i.e. CD8 mRNA expression and CD8^+^ expressing T cells) was verified by the time-dependent ROC curve and Kaplan–Meier survival estimates

**Figure 3:**
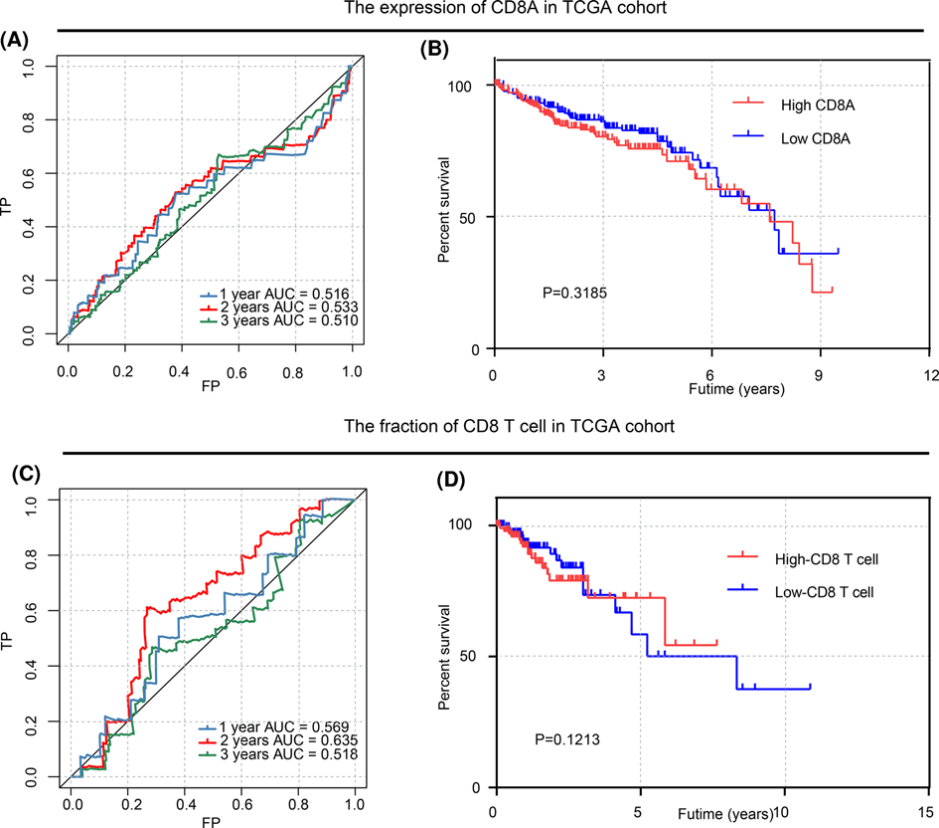
Time-dependent ROC curve and analysis of Kaplan–Meier survival estimates for *CD8* mRNA expression and CD8^+^ T cells (**A**) Time-dependent ROC curve for the expression of CD8A in TCGA cohort. (**B**) Analysis of Kaplan–Meier survival estimates for the expression of CD8A in TCGA cohort. (**C**) Time-dependent ROC curve for the fraction of CD8 T cell in TCGA cohort. (**D**) Analysis of Kaplan–Meier survival estimates for the fraction of CD8 T cell in TCGA cohort.

To validate the prognostic ability of the immunocyte fraction, we used *CD8* mRNA expression (461 cases in TCGA) and CD8^+^ expressing T cells (216 cases in TCGA) in CRC patients for the time-dependent ROC curve and the analysis of Kaplan–Meier survival estimates. The areas under the curves (AUCs) of *CD8* expression were 0.516, 0.533, and 0.510 for 1-, 2-, and 3-year survival times, respectively, as shown in Figure 3A. The high *CD8* expression group and the low *CD8* expression group were divided by an optimal cut-off value of 35, which was generated by the survminer R package. However, there was no significant difference between the high expression group and the low expression group in the Kaplan–Meier survival estimates by the log-rank test (*P*=0.3185) (Figure 3B). Furthermore, the same methods were used to verify the predictive ability of the fraction of CD8^+^ T cells. The AUCs of the CD8^+^ T cell fractions were 0.569, 0.635, and 0.518 for 1-, 2-, and 3-year survival times, respectively, as shown in Figure 3C. In Figure 3D, no statistically significant differences between low CD8^+^ fraction group and the high CD8^+^ fraction group were shown in the Kaplan–Meier survival estimates by the log-rank test (*P=*0.1213). *A novel prognostic immunoscore was structured by LASSO regression*.

**Figure 4:**
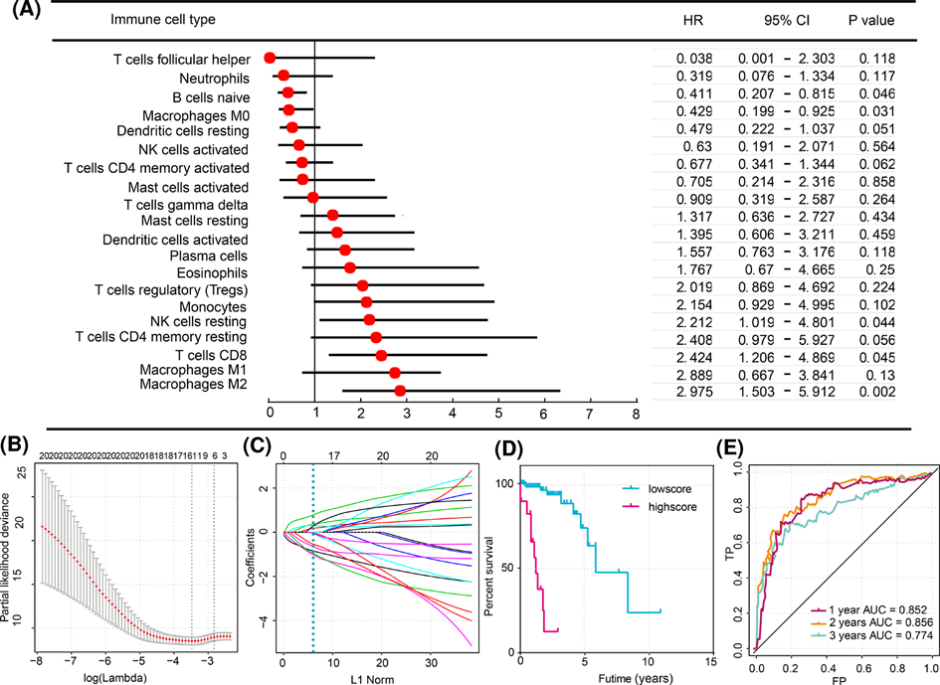
Structure of the immunoscore model (**A**) The relationship between different immunocyte subtypes and overall survival in the training cohort was revealed in the forest plot. (**B**) Unadjusted HRs are shown with 95% confidence intervals. (**C**) LASSO coefficient profiles of the proportions of 20 immunocyte subtypes. The dotted line indicates the value chosen by tenfold cross-validation. Tenfold cross-validation for the selection of the tuning parameter in the LASSO model. The partial likelihood deviance is plotted against log (λ), where λ is the tuning parameter. Partial likelihood deviance values are shown, with error bars represented as s.e. The dotted vertical lines are drawn at the optimal values by minimum criteria and 1 − s.e. criteria. In (B,C), the values above the graphs represent the number of cell types involved in the LASSO model (**D**). KaplanδMeier curves for overall survival based on the immunoscore in the training cohort (**E**). Immunoscore assessed by the time-dependent ROC curve in the training cohort. The areas under the ROC curve for the immunoscore were 0.852, 0.856, and 0.774 at 1-, 2-, and 3-year survival times, respectively.

The survminer R package was applied to calculate the optimal cut-off value for the proportion of each immunocyte subtype in the training group (131 patients) (Table 2). Because the fractions of memory B cells and naive CD4^+^ T cells were too small to calculate the cut-off values, they were excluded from the following study. When the proportion of one type of cell was less than the optimal cut-off value, a value of 0 was assigned, and a value of 1 was assigned otherwise. Using these values, we generated a forest plot to disclose the relationship between the immunocyte fraction and overall survival, as shown in Figure 4A.

**Table 2:** The optimal cut-off value for each cell calculated by survminer package

Cell type	Cut-off value
B cells naive	0.017534483
B cells memory	-
Plasma cells	0.010740751
T cells CD8	0.101560476
T cells CD4 naive	-
T cells CD4 memory resting	0.230188921
T cells CD4 memory activated	0.002619793
T cells follicular helper	0.054051116
T cells regulatory, Tregs	0.075296913
T cells γδ	0
NK cells resting	0.044819172
NK cells activated	0.041832158
Monocytes	0.006696936
Macrophages M0	0.107594405
Macrophages M1	0.111777439
Macrophages M2	0.064314625
Dendritic cells resting	0.017116027
Dendritic cells activated	0.00410661
Mast cells resting	0.023567448
Mast cells activated	0.096627215
Eosinophils	0.02029439
Neutrophils	0.020678702

To build a novel and accurate prognostic immunoscore, we applied LASSO regression to simplify and regularize the immunoscore for CRC patients. The immunoscore was calculated using the following formula: immunoscore = B cells naive × (−0.85397923) + plasma cells × 0.177521701 + T cells CD8 × (−0.792726373) + T cells CD4 memory activated × (−0.079212861) + T cells follicular helper × (−1.023964239) + T cells γδ × (−0.038245466) + NK cells resting × 0.363350632 + monocytes × 0.302307974 + macrophages M0 × (−0.656014902) + dendritic cells resting × (−0.970563582) + mast cells activated × 0.041272045 + eosinophils × 0.297516302 + neutrophils × (−0.547332907). In the formula, the immunocyte subtype level was set at 0 or 1 by the survminer R package, which generated the optimal cut-off value for each immunocytes subtype. Figure 4B shows a generalized cross-validation plot of the immunocyte fractions in the training cohort. The partial likelihood deviance was plotted against log (Lambda, λ), where λ was the tuning parameter. The dotted vertical lines were drawn at the optimal values using minimum criteria and 1 − s.e. criteria. The suitable λ occurred when the model included 13 variables. Moreover, we analyzed the risk score in different group in Table 3. Patients in stage III–IV have higher risk score than stage I–II group. We provided the specific clinical information and risk score in the supplementary materials.

**Table 3:** The risk score in different groups

Variable	Group	Score	*P*-value
Age	<65	−1.13 ± 0.99	0.241
	≥65	−1.29 ± 0.87	
Gender	Male	−1.13 ± 0.88	0.308
	Female	−1.26 ± 1.01	
Stage	Stage I–II	−1.33 ± 0.98	0.034
	Stage III–IV	−1.04 ± 0.88	
Lymphatic invasion	Positvie	−1.14 ± 0.83	0.634
	Negative	−1.21 ± 1.01	

Figure 4C shows the estimated coefficients from the LASSO-derived model, and the dotted line indicates the coefficient chosen by cross-validation in the training cohort. The patients of the training cohort were assigned to either the high or low immunoscore group based on the optimal cut-off value. Notably, patients of the high immunoscore group had a significantly lower overall survival than those of the low immunoscore group (*P*<0.01). Moreover, the prognostic accuracy of the immunoscore model was evaluated by computing the AUC of the time-dependent ROC curve in the training cohort. Higher time-dependent AUC represented better performance and stability for the model. The AUCs of the model were 0.852, 0.856, and 0.774 for 1-, 2-, and 3-year survival times, respectively. Taken together, these results show that the immunoscore possessed good specificity, sensitivity, and time-dependent stability for predicting the overall survival of CRC patients.

### The effectiveness of the immunoscore for predicting overall survival and disease-free survival was verified in the validation cohort and entire cohort

**Figure 5:**
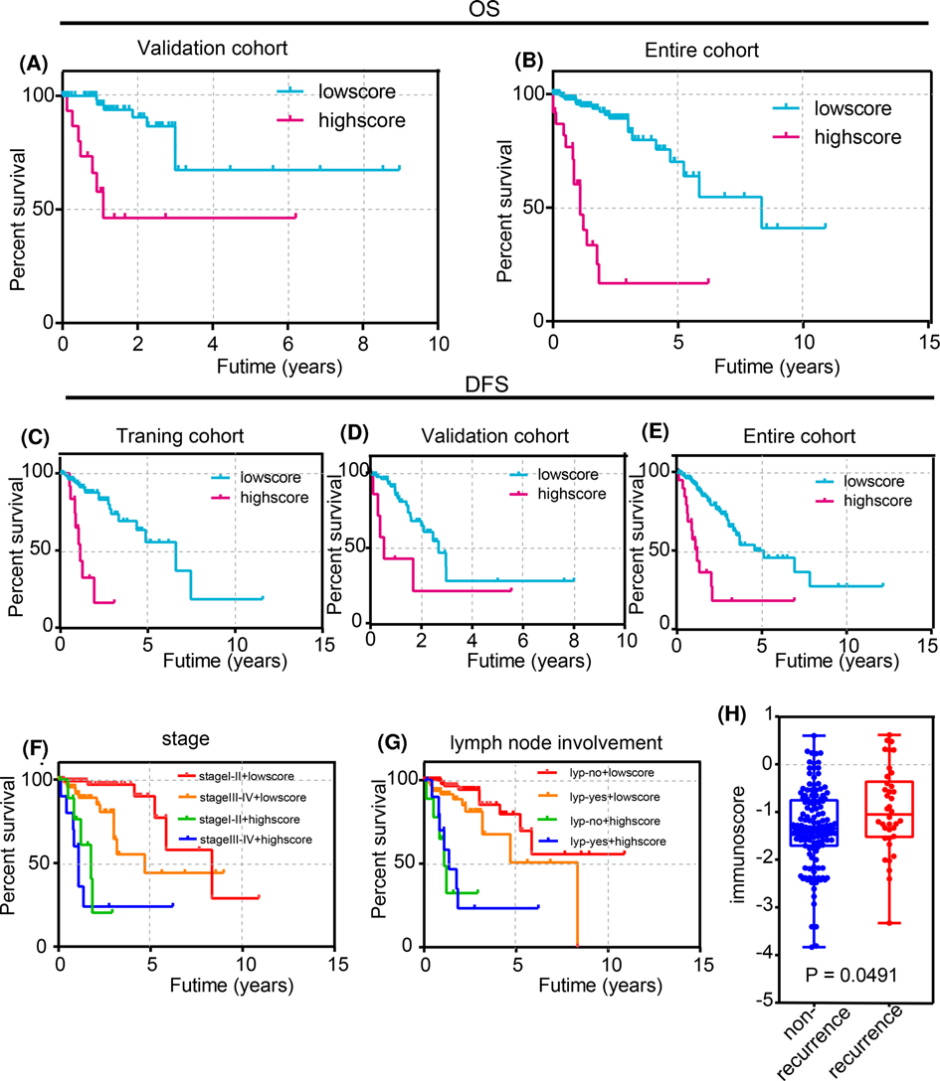
Validation of the immunoscore model (**A**,**B**) Kaplan–Meier curves for overall survival based on the immunoscore in the validation cohort and entire cohort. (**C–E**) Kaplan–Meier curves for disease-free survival based on the immunoscore in the training cohort validation cohort, and entire cohort. (**F**) Joint effects of overall survival stratified by the immunoscore and CRC clinical parameters (stage). (**G**) Joint effects of overall survival stratified by the immunoscore and CRC clinical parameters (lymph node involvement). (**H**) A significant difference was observed between the blue and red curves in all four profiles (*P*<0.05). The different immunescore between the recurrence group and the non-recurrence group (*P*<0.05).

To validate the prognostic ability of the immunoscore in different patients, we applied the formula of the immunoscore to the validation cohort (87 patients) and the entire cohort (218 patients). The patients were divided into high and low immunoscore groups using the optimal cut-off value calculated by the survminer R package. Not surprisingly, patients of the high immunoscore group tended to have a lower overall survival than patients of the low immunoscore group in both the validation cohort and entire cohort, which was similar to the trend of the training cohort (Figure 5A,B). In addition, we compared disease-free survival between the high immunoscore group and the low immunoscore group in the training cohort, validation cohort, and entire cohort, as shown in Figure 5C–E. Consistent with the previous findings, the patients of the low immunoscore group had higher disease-free survival in the training cohort, validation cohort, and entire cohort, which showed great prognostic ability of the immunoscore for CRC patients.

### The immunoscore was combined with pathological characteristics to improve the prognostic ability

According to current knowledge, the stage and the status of lymphatic metastasis are usually applied to determine the prognosis of CRC patients. We performed univariate and multivariate Cox regression analyses on this dataset to assess the independent predictive ability of the prognostic model. Univariate Cox regression results showed significant differences when the patients were grouped by ‘stage’, ‘lymph node involvement’, and ‘immunoscore model’ predictors (*P*<0.05). However, sex, age, and TMB did not correlate with overall survival (*P*>0.05). Subsequently, we included the factors in a multivariate Cox regression analysis (Table 4), which revealed that the ‘stage’, and ‘immunoscore model’ variables were independent prognostic factors related to overall survival (*P*<0.05). We did not include lymph node metastatic status in the multivariate analysis, considering that the stage classification already included it. Therefore, we combined the immunoscore with the aforementioned pathological characteristics to refine its predictive ability using a Kaplan–Meier curve in the entire cohort. As shown in Figure 5F,G, patients belonging to the red curve possessed a remarkably good prognosis compared with those of other groups. In addition, the immunoscore was different between the tumor recurrence group and the non-recurrence group according to the independent-samples *t* test (*P*<0.05), as shown in Figure 5H. Therefore, combining the immunoscore with the pathological characteristics can improve the prediction of prognosis in CRC patients, which has the value to be used as a reference for the individualized regimens on CRC patients and can, to some extent, direct the treatment protocol.

**Table 4:** The correlations of immunoscore with patients’ overall survival in CRC based on TCGA dataset using univariate and multivariate Cox analyses

Variable	Univariate Cox		Multivariate Cox	
	*P*-value	HR (95% CI)	*P*-value	HR (95% CI)
Gender (male vs female)	0.508	1.259 (0.637–2.486)	-	-
Age (>65 vs ≤65)	0.781	1.106 (0.545–2.245)	-	-
Stage (III–IV vs I–II)	0.02	2.312 (1.139–4.692)	0.013	2.072 (1.569–3.651)
TMB (high vs low)	0.238	1.560 (0.746–3.265)	-	-
Prognostic immunoscore (high risk vs low risk)	<0.001	9.219 (4.571–18.595)	<0.001	8.080 (3.635–17.964)

Abbreviation: CI, confidence interval.

### The relationship between the TMB score and immunocyte fraction was confirmed by TCGA data, and the poor outcome of patients in the high TMB group could be identified by the novel immunoscore

**Figure 6:**
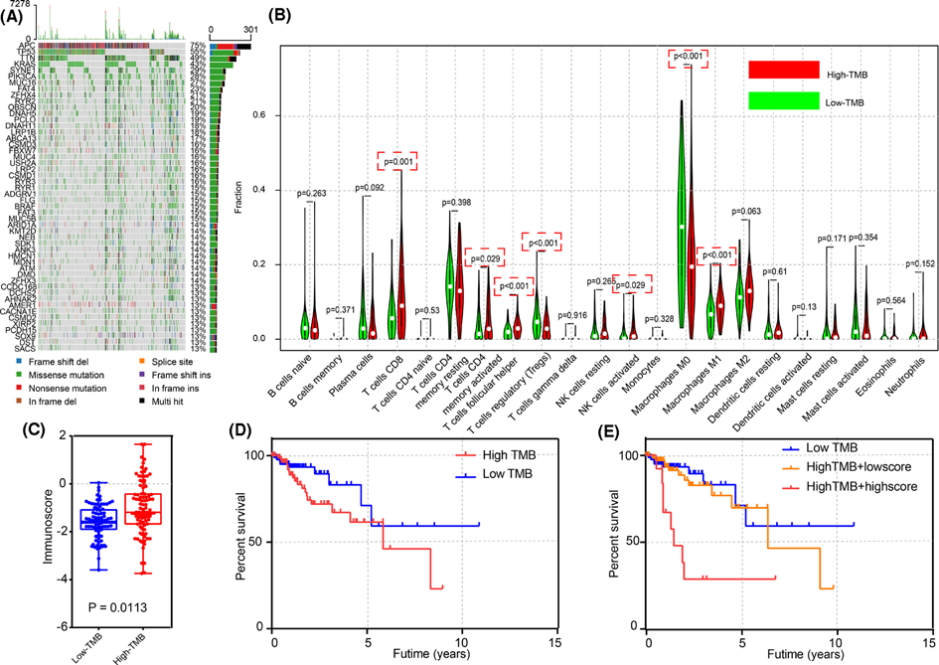
Mutations in CRC patients, and the distribution of immunocytes based on different TMB scores (**A**) A waterfall plot of the top 50 mutated genes in CRC patients. (**B**) Different immunocyte fractions in high and low TMB groups. CD8^+^ T cells, memory activated CD4^+^ T cells, follicular helper T cells, regulatory T cells, activated NK cells, M0 macrophages, and M1 macrophages had a *P*-value <0.05. (**C**) A difference in the immunoscore between high and low TMB groups (*P*=0.0113). (**D**) Kaplan–Meier curves for overall survival based on the TMB group (*P*>0.05). (**E**) The ability of the immunoscore to identify poor prognostic patients in the high TMB group is shown in (E).

Mutation data of the CRC patients were obtained from TCGA by the maftools package. As shown in Figure 6A, APC regulator of WNT signaling pathway (*APC*), tumor protein p53 (*TP53*), and titin (*TTN*) were frequently mutated in CRC patients, which is consistent with the results of previous studies [15]. On the basis of the mutation data, we calculated the TMB score for each CRC patient. The CRC patients were then divided into high and low TMB groups by the optimal cut-off value. Surprisingly, there were differences in immunocyte subtypes and the immunoscore between high and low TMB groups according to the independent-samples *t* test, as shown in Figure 6B,C. However, there was no significant difference in overall survival between high and low TMB groups. As shown in Figure 6D,E, with the help of the immunoscore, we could identify patients in the high TMB group with relatively poor prognoses, and these patients may be suitable for immunotherapy.

### The correlation between the immunoscore and the mRNA expression of selected immune checkpoints was verified by the Pearson correlation coefficient and scatter plots

**Figure 7:**
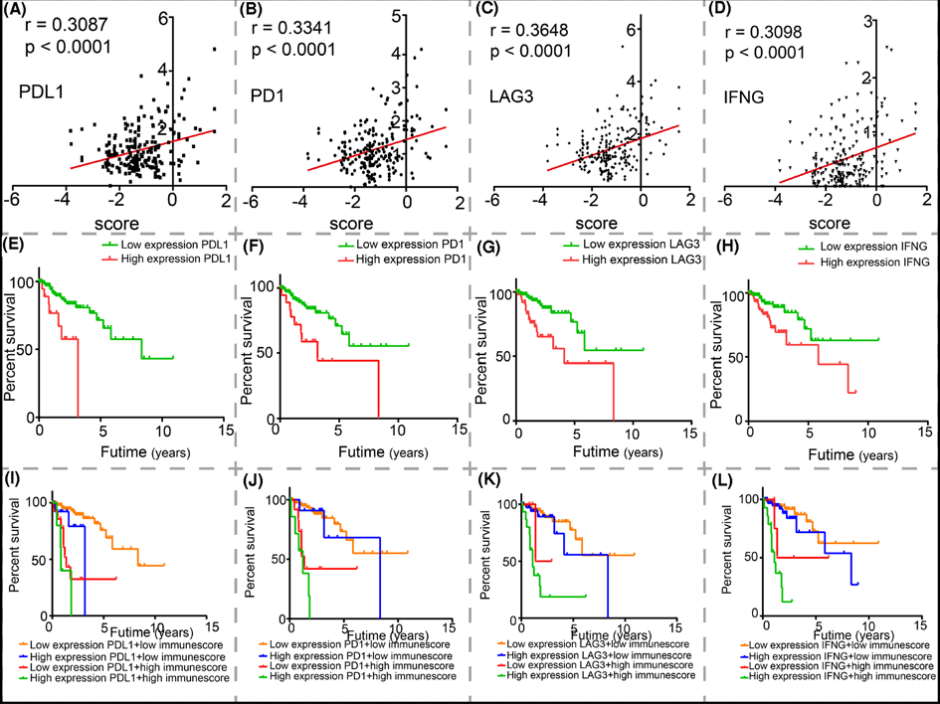
The relationship between the immunoscore and immue checkpoints (**A–D**) Relationship between the immunoscore and immune checkpoints [(A) PD-L1, (B) PD-1, (C) LAG3, and (D) IFNG] was tested by Pearson correlation analysis. (**E–L**) Joint effects of overall survival were stratified by the immunoscore and the expression of immune checkpoint genes.

With the use of bioinformatics tools, we could easily obtain the expression levels of important immune checkpoints in CRC patients from TCGA data. Therefore, we investigated the relationship between these immune checkpoints and the immunoscore. Surprisingly, there was a significant positive correlation between the immunoscore and the selected immune checkpoints, including *PD-1*, *PD-L1*, *LAG3*, and *IFNG*. The positive correlation is represented by the Pearson correlation coefficient and scatter plots, as shown in Figure 7. Furthermore, we combined the expression levels of the positively related genes with the immunoscore to understand the significance of the novel immunoscore in the K-M curves. The optimal cut-off values of these genes were calculated by the survminer R package. Among the four positively related genes, the low immunoscore and low expression of the PD-L1 group showed more advantages than the other groups. Moreover, the patients belonging to the green curve possessed a remarkably poor prognosis, which should be pointed out to clinicians.

## Discussion

In the present study, we developed and validated a novel immunoscore for 13 immunocyte subtypes to improve the prediction of CRC patient survival. The results showed a clear separation of overall survival and disease-free survival curves between the patients with high and low immunoscores. Patients with high immunoscores were predisposed to poor prognoses. If the immunoscore was used in clinical practice, clinicians could adjust the treatment plan according to the results to generate an individualized and comprehensive protocol for each CRC patient. In addition, the combined use of the immunoscore and pathological characteristics increased the reliability of the predicted prognosis.

Previous studies have routinely used immunohistochemical techniques to study different immunocyte fractions [16–18]. However, due to the inherent shortcomings of the technique, results were always derived from a small sample size or a few cell subtypes [19]. In contrast with previous studies, we used the newly developed algorithm CIBERSORT to characterize immunocyte fractions based on the high-throughput gene expression profile from TCGA. A previous study also used the CIBERSORT algorithm for analysis to develop an immunescore prognostic model for immune-related genes associated with TP53 mutation status, and have validated their relationship with prognosis in CRC patients [20]. They also used two public datasets, notable for their discovery of prognostic immune-related genes associated with specific gene status—TP53, and collected them to create an immune prognostic model. The results of their analysis were similar to ours, with a significant relationship between immunescores and OS in the TP53 subgroup—high group of OS rates are worse than the lower group. It is worthwhile to learn from them that they established a nomogram based on multivariate Cox regression coefficients of immunoscore, TP53 status, tumor stage, tumor location, and microsatellite status, which is a better predictive model for prognosis than immunoscore or TNM staging. We will continue to watch for follow-up information to be uploaded for future addition to our analysis. This approach allows researchers to obtain a better understanding of the cellular immune response and to characterize more cell subtypes in a larger cohort than was previously possible [21]. LASSO-Cox regression was further used as a statistical method to structure the immunoscore model, which significantly improved the predictive accuracy. The prognostic ability of the novel immunoscore was verified in the validation cohort, as well as in the entire cohort, and the results indicated that the immunoscore had excellent stability and accuracy. When we combined the immunoscore with the TNM stage and other pathological characteristics, the prognostic ability of the immunoscore was reinforced. In a recent study [22], the TMB score was used to predict immunotherapy effectiveness, and it has become an important biomarker across many cancer types to identify patients that will benefit the most from immunotherapy. In the present study, we found that the immunoscore could identify patients in the high TMB group with a poor prognosis, specifically those who may benefit the most from immunotherapy. In addition, our results showed a positive correlation between the immunoscore and the mRNA levels of *PD-1*, *PD-L1*, *LAG3*, and *IFNG* [23–27], which indicated the accuracy and reasonability of the immunoscore for CRC patients.

However, the present study had several limitations. Firstly, the present study made use of a public dataset, and we could not obtain all the information that we needed, such as the recognized immune-related MSI status, the patient’s albumin level, lymphocyte count, nutritional status, and other characteristics, We therefore did not perform the relevant analysis, which may have caused systematic errors. Besides, the site of recurrence in patients and T-cell infiltration was also not found in UCSC, but we would like to collect case samples and follow-up data in future research. Secondly, we simply validated the accuracy of the immunoscore in the validation cohort rather than in our own clinical samples. Thirdly, our reseach just compared the prognostic ability between the CD8^+^-expressing T cells model and immunoscore model, which means that the comparative research between other variable remains to be done. Above all, the mechanism by which the prognostic immunoscore affects CRC requires further investigation. In conclusion, we developed a novel prognostic immunoscore that is a reliable predictor of overall survival and disease-free survival of CRC patients, which can improve the clinical management of patients with this disease.

## Supplementary Material

Supplementary Materials

## Data Availability

The datasets used and analyzed during the current study are available from the corresponding authors on reasonable request.

## Abbreviations

AUC: area under the curve
CRC: colorectal cancer
LASSO: least absolute shrinkage and selection operator
ROC: receiver operating characteristic
TCGA: The Cancer Genome Atlas
TMB: tumor mutational burden
UCSC: University of California at Santa Cruz Xena browser
